# *Caveat emptor* NICE: biased use of cost-effectiveness is inefficient and inequitable

**DOI:** 10.12688/f1000research.7191.1

**Published:** 2015-10-16

**Authors:** Jack Dowie, Mette Kjer Kaltoft, Jesper Bo Nielsen, Glenn Salkeld

**Affiliations:** 1 Faculty of Public Health and Policy, London School of Hygiene and Tropical Medicine, London, UK; 2 Odense University Hospital, Svendborg, Denmark; 3 Research Unit for General Practice, Department of Public Health, University of Southern Denmark, Odense, Denmark; 4 Faculty of Medicine, University of Sydney, Sydney, Australia

**Keywords:** cost-effectiveness, incremental cost-effectiveness, decremental cost-effectiveness, south-west quadrant

## Abstract

Concern with the threshold applied in cost-effectiveness analyses by bodies such as NICE distracts attention from their biased use of the principle. The bias results from the prior requirement that an intervention be effective (usually 'clinically effective') before its cost-effectiveness is considered. The underlying justification for the use of cost-effectiveness as a criterion, whatever the threshold adopted, is that decisions in a resource-constrained system have opportunity costs. Their existence rules out any restriction to those interventions that are 'incrementally cost-effective' at a chosen threshold and requires acceptance of those that are 'decrementally cost-effective' at the same threshold. Interventions that fall under the linear ICER line in the South-West quadrant of the cost-effectiveness plane are cost-effective because they create net health benefits, as do those in the North-East quadrant. If there is objection to the fact that they are cost-effective by reducing effectiveness as well as costs, it is possible to reject them, but only on policy grounds other than their failure to be cost-effective. Having established this, the paper considers and seeks to counter the arguments based on these other grounds. Most notably these include those proposing a different threshold in the South-West quadrant from the North-East one, i.e. propose a 'kinked ICER'. Another undesirable consequence of the biased use of cost-effectiveness is the failure to stimulate innovations that would increase overall health gain by being less effective in the condition concerned, but generate more benefits elsewhere. NICE can only reward innovations that cost more.

## Introduction

The publication of the Claxton report containing an estimate of the willingness to pay for an incremental Quality-Adjusted Life Year (QALY) implicit in the expenditure patterns of the NHS of England and Wales has refocused attention on the use by the National Institute for Care and Health Excellence (NICE) of cost-effectiveness as one criterion in its reimbursement decisions ^1, 2^. The suggestion that the empirical threshold for cost-effectiveness is about £13,000 (but probably lower), compared with the £20,000 to £30000 range acknowledged by NICE, assumes greater significance in the context of the growing adoption of the NICE model, or some version of it, in other jurisdictions facing the same challenges. The introduction and use of the formal and relatively transparent NICE process has undoubtedly been a major advance, compared with the situation in countries who are in official denial about the need to prioritise and do so with some transparency. The idea that any resource-constrained health or other public service can function efficiently and equitably - and hence ethically - without employing cost-effectiveness as a key principle we take to be absurd. *How* the principle - which would be better called *opportunity cost*-effectiveness - is implemented, is the only issue. There are numerous valid and important debates to be had on this, including the one that concerns us here.

The NICE advance has been bought at the price of biased use of the principle of cost-effectiveness and, as a corollary, biased support for innovative technologies. These biases are built into its legal obligations. NICE is, formally speaking, an independent ‘non-governmental public body’ whose remit comes from the Department of Health, which funds it. That remit is to appraise the clinical and cost-effectiveness of technology x within its licensed indication for treating disease y. To be considered in the scoping process for possible appraisal, the technology must be 'either new or an innovative modification of an existing technology with claimed benefits to patients or the NHS judged against the comparator(s).' The purpose of the NICE appraisal is to decide whether the new technology works well (is clinically effective) *and* good value for money (is cost-effective). At no stage of the scoping or appraisal process is an innovation that claims to be cost-effective and 'good value for money', but not 'clinically effective' in relation to the comparators, eligible for consideration. So there is no point in their being put forward. Formally, the ban on such innovations is imposed on NICE from above, but there has never been any indication that the organisation is other than in full agreement with it, and accordingly with the biased use of the cost-effectiveness principle involved in prior filtering by clinical effectiveness.

This is not an empirical issue. The major project by Claxton and colleagues has yielded important insights into the cost-effectiveness threshold implicit in the behaviour of the NHS, establishing the average cost of an extra QALY generated (conservatively put at £12,396), the number of QALYs likely to be forgone as a consequence of approving a more expensive technology, and where those QALYs are likely to be lost in its 23 broad programme budget categories. The authors claim that this explicit quantification of the scale of opportunity costs the NHS faces provides a basis for determining the appropriate threshold for NICE decisions, as well as those made centrally by the NHS and Department of Health.

For those concerned with the inadequacies of the QALY as an effectiveness measure, the report emphasises that the estimation methods can cope with other outcomes, such as patient-reported outcome measures (PROMs), subject to their being brought within the opportunity cost framework ^3^. The methods can also be extended to allow weights to be attached to the type of health that is forgone.

However, neither this impressive empirical progress, nor the subsequent debate ^4, 5^ impinge on the present argument concerning the biased application of the cost-effectiveness principle. This empirical advance will simply make it easier to establish the displacement consequences of new cost-effective innovations, wherever and however they occur. As has been the case since the founding of NICE, the report and discussion ignores the mammoth standing silently in the south-west corner of the policy room: the proper use of cost-effectiveness as a criterion.

From its inception NICE has never adopted the principle of cost-effectiveness, only the censored version of it called *incremental* cost-effectiveness. The Claxton report accepts this corruption of the principle, the single peripheral mention of *decremental* cost-effectiveness being buried under the heading 'multiple thresholds' in an Appendix. As independent analysts, they might be expected to state, upfront in one sentence, that it is in the light of the NICE remit that they exclude from consideration any intervention which is cost-effective by being less effective, but less costly.

The objective in section 2 below is to end the sinister bifurcation of the single and unified cost-effectiveness principle. Separating incremental and decremental cost-effectiveness is as meaningful as separating right-handed and left-handed ambidexterity. It may be helpful for operational reasons to characterise the differing origins of cost-effectiveness, but the two cannot be separated for policy purposes without abandoning the principle.

In section 3 we present and seek to counter the main arguments against accepting and promoting innovations that fall in the South-West (SW) quadrant of the cost-effectiveness plane and under a linear Incremental Cost-Effectiveness Ratio (ICER).

One of the most powerful reasons for the individual citizen to favour a National Health Service will be its rationality from a Rawlsian perspective. Under great uncertainty (approximating a 'veil of ignorance') as to what diseases and conditions oneself, one's children, grandchildren and significant others will suffer from in the future, the greater the reason to support the consistent application of the principle of cost-effectiveness throughout the system. And hence the greater the reason for bodies making decisions within it to treat South-West innovations in exactly the same way as North-East ones, using the same threshold ^10^.

## The integrity of the cost-effectiveness principle

**Figure 1.**
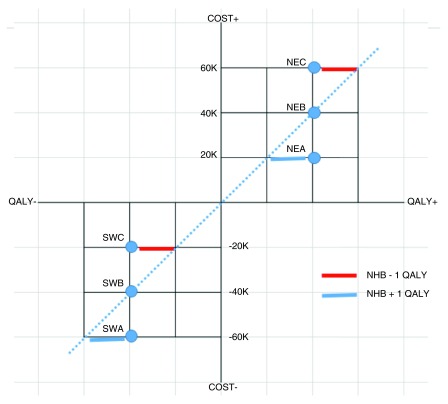
Full cost-effectiveness plane based on Claxton’s figure ^1^ confined to North-East quadrant.

We believe we can achieve our aim quickly and simply, by taking the key diagram in Claxton, confined to the North-East (NE) quadrant of the cost-effectiveness plane, and extending it to include all its four quadrants ( Figure 1). The original figure in Claxton implicitly acknowledges the existence of the SW quadrant by extending the dotted ICER, or threshold, line for a short distance into it, doing so without distortion or kink ^1, 2^. (A kinked threshold line, steeper in the SW quadrant than in the NE, is one of the main arguments considered in part 2.) We can leave the South-East and North-West quadrants empty, as having dominated solutions that make the argument here irrelevant. Any new technology in the SE quadrant should be adopted as cost-effective and, because it both costs less and is more effective, trumps any other intervention beneath the ICER line in either the NE or SW quadrants. (It will often be referred to as ‘cost-saving’ rather than ‘cost-effective’ by those whose attention is restricted to the eastern hemisphere.)

Essentially we duplicate the Claxton diagram, rotate the duplicate through 180 degrees and place it in the SW quadrant. And relabel appropriately. For the text explanation accompanying the Claxton diagram and our translation of it for the SW quadrant, see the Appendix.

All we intend, and need, to show in this section, is that interventions NEA and SWA are equally cost-effective, both resulting in a Net Health Benefit increase of 1 QALY. NEA adds 2 and loses 1. SWA adds 3 and loses 2. Case made. QED.

NEB (+2, -2) and SWB (-2, +2) are both neutral, involving equal gain and loss.

Neither NEC (+2, -3) or SWC (-2, +1) is cost-effective, with loss exceeding gain.

Any intervention below the dotted ICER line is cost-effective – assuming it is not dominated, i.e. there is not an intervention that is further south and at least as far to the east, or further east and at least as far to the south.

It is important to use consistent terminology throughout the plane. There is much talk about 'disinvestment' in the threshold debate, with the QALY lost by investing in NEA characterised as such. But SWA also represents an investment in new technology and the two QALYs lost as a result are the disinvestment resulting from this new investment.

This diagram, like any cost-effectiveness plane or analysis, assumes a particular threshold. But it should be clear that the slope of the ICER (whether the threshold is 13k, 20k or 30k), how much uncertainty surrounds any empirical calculation of it, and where displacement or disinvestment specifically occurs - are all irrelevant to the present argument. The diagram simply confirms that the principle of cost-effectiveness, justified either on efficiency or ethical grounds, requires its implementation in unbiased form, treating incremental and decremental origins as equally valid. If censoring is undertaken, it should be explicitly acknowledged as representing the abandonment of the cost-effectiveness principle and justification sought on other grounds.

It will be obvious that the lower the threshold, the smaller the area under the ICER line in the NE quadrant and the greater the area under it in the SW quadrant; hence the greater the scope for interventions to be developed within the latter. At the limit, if the threshold were approaching at, or approaching zero, all interventions in the SW quadrant would be cost-effective, and none in the NE.

Having rejected the principle of cost-effectiveness as the basis for ruling out SW innovations, what other grounds might be advanced against adopting or encouraging them?

## Go South West? The arguments and counter-arguments

*“SW interventions are simply wrong because they take away from them something people already have.”*

The simplest argument against treating the SW and NE quadrants in the same way boils down to the rights-based objection that adopting a SW intervention would involve 'taking away' effectiveness (in this illustration, QALYs) from individuals who currently enjoy it. No benefits to others can justify this breach of rights, it is said. But this principle, even if it were to be agreed that *current* recipients would not be forced to move on to the less effective treatment because it is now the cost-effective one, lacks any justification when extended to those who acquire the same condition *in the future.* Having never enjoyed the effectiveness of the old treatment, they cannot have a right to it taken away from them. Those who become ill later cannot ethically be favoured, simply because they suffer from this disease or condition, rather than from some other one. The Rawlsian rationality of this social ethic, even from an individual perspective, is clear.

‘… the rational Rawlsian patient – who does not yet know whether they will personally suffer from condition X, … or, instead, from any of the wide range of other possible conditions – should clearly favour the wider distribution of benefits that comes from applying the decision rule consistently in the SW as well as NE quadrant.’ ^6^ p.457

*“SW interventions will produce ill health which will require treatment and impose extra costs”*

Gandjour ^7^ argues that the experience of loss, or even anticipation of loss, can have negative health consequences of various sorts. Unfortunately apart from the individual focus of his example ^8^, Gandjour fails to address the key issue regarding intervention for any ‘lossaversionitis’ resulting from the introduction of SW interventions. Consistency and equity demands that realistic interventions for lossaversionitis go into the cost-effectiveness analysis, along with all other interventions. So, while the illness created may be real, there is no guarantee it will be treated. Prevention of lossaversionitis may be the optimal strategy.

“ *SW interventions should not occur unless it can be shown that there will be a net increase in health*”

Sendi, Gafni and Birch’s challenge to the SW argument helps clarify an important point as to why we adhere to it and reject their alternative ^9^. They point out that there is no guarantee that the amount of resources released by a specific SW intervention will result in a net increase in QALYs. This will occur only if the resources are diverted to an intervention that will achieve this and not every intervention below the ICER line will do so. Correct. But the inability to determine specifically where the resources are diverted from to fund a new intervention in the NE quadrant is also unknown. So fundamentally their objection is to the use of an 'overall subjective ICER threshold’ for the NE, not just the SW. Their alternative approach involves use of a ‘decision maker’s plane’, where a specific intervention replaces a specific intervention only if the effect on overall health gain is positive. This is simply not the real world of any national health service, let alone the NHS, as pointed out by Claxton and colleagues:

‘NICE cannot be expected to reflect what is likely to be marked variation between local commissioners and providers in how they react to an effective reduction in their budget as a result of positive guidance. Given NICE’s remit, it is the expected health effects (in terms of length and QoL) of the average displacement within the current NHS (given existing budgets, productivity and the quality of local decisions) that is relevant to the estimate of the threshold.’ ^2^ p.8

We see no justification for imposing higher requirements of specificity regarding displacement on SW interventions than on NE ones.

*“Some SW interventions are acceptable, but only those under a (very) kinked ICER”*

Some see validity in the SW argument but wish to restrict its application. The main mechanism suggested is a 'kinked' ICER - a threshold line which is steeper in the SW quadrant than it is in the NE one ^10^. The slope in the SW quadrant should reflect the ‘acceptable’ Willingness to Accept/Willingness to Pay (WTA/WTP) ratio. This will be greater than 1, hence the steeper slope. Along similar lines, Kent, *et al.* suggest establishing a Maximally Acceptable Difference (MAD) in an ‘acceptability trial’ for SW interventions ^11^, the MAD being ‘a level of inferiority beyond which a new less expensive agent would no longer be attractive when compared to the best standard.’

The most frequent objection to the SW argument is that attitudes to loss and gain (WTA and WTP) are asymmetric, with WTA typically higher or much higher than WTP, because of 'loss aversion'. While income and other factors play some role, the dominant explanation offered for such loss aversion is the so-called ‘endowment effect'. 'We' regard losing a specified amount of what we already possess as proportionately worse than gaining that same amount and require greater compensation to accept the loss than we would pay for an equal size gain.

Numerous empirical studies have confirmed loss aversion as descriptively true *at the individual* and *aggregated individual level*, so this is not in dispute. Nor is the fact that the WTA/WTP ratio varies from situation to situation. In an example particularly relevant for this paper

… the farther a good is from being an ordinary private good, the higher the ratio…. Ratios are highest for health/safety and public/non-market goods, next highest for ordinary private… The closer the good comes to being actual money, the smaller the ratio ^12^. pp.434–5

Grutters *et al.* ^13^ found that using a WTA and a WTP format for the cost attribute in a discrete choice experiment (on transferring elements of hearing aid provision from the medical sector to private hearing aid dispenser) elicited different preferences and monetary values . They concluded

Most discrete choice experiments in health care use the concept of WTP, but WTA has also been used… to our knowledge, no study has paid explicit attention to when the cost attribute should be defined as a payment or a discount. The lack of clarity on how to address the disparity between WTA and WTP in discrete choice experiments probably results from the fact that before the present study, the disparity had not yet been examined… ^13^ p.1118

The case for adopting a SW intervention becomes progressively stronger as the saving from the loss of a QALY increases. If there is a way one can save 60k rather than 30k by giving up a QALY, then the benefits generated elsewhere are doubled. But whether a SW intervention is cost-effective always depends on the ICER.

In a pharma-sponsored study Liew, *et al.* calculated that shifting patients from their atorvastatin to simvastatin would lead to a net cost saving of €131 per subject, but also a loss of 0.03 quality-adjusted life-years (QALYs) per subject ^14^. These equated to a decremental cost-effectiveness ratio of €4,777 per QALY lost. The authors’ conclusion that ‘It would be cost effective to maintain patients on atorvastatin for primary prevention rather than switch them to simvastatin’ is valid, given the threshold is set above €4,777.

In an example relating to a new intervention for pain management, Soares and Dumville report a decremental ratio of £1,220, going on to show in a Cost Effectiveness Acceptability Curve analysis that this would be cost-effective only at very low thresholds ^15^. The authors leave it ambiguous as to whether the decision rule (threshold) they rightly say is required in both NE and SE quadrants should be the same one. We maintain that the principle of cost-effectiveness requires that they be the same and that no logical or ethical case can be made for any kinked ICER in a public system ^16^. We question the relevance of aggregated asymmetric individual preference results to group level policy making, in the context of a resource-constrained system committed to equitable efficiency. The fact that the Grutters study not only produced different ratios for 'gainers' and 'losers', but that the two sets of results also depended on how the cost attribute was framed, confirms to us that permitting this ratio to be other than 1 is unethical at a societal level. Searching for the conditions under which one or other framing should be used, which they contemplate, is inappropriate, since an equitable public policy requires an unbiased single estimate of WTP&A.

Establishing that single value becomes the research challenge. Whether it will result in a ICER near the current NE one is unknown, because stated community preferences have never been investigated under the appropriate, Rawlsian, conditions of complete uncertainty as to where the investment and disinvestment will fall, and hence complete uncertainty about the future personal implications for the respondent.

*“Prospect theory and psychic numbing are legitimate bases for public policy”*

Descriptive theories of decision making, such as prospect theory, claim that individuals do not maximise expected value or utility, instead treating probabilities as non-linear and having value functions that are concave for gains and convex for losses ^17^. This may or may not be true at the individual level, but if is, to be used as the basis for rejecting SW policy interventions, transportation from the individual to society needs to be regarded as legitimate. In what Featherstonhaugh, Slovic and others refer to as ‘psychic numbing’ and the ‘collapse of compassion’, the value of a life-saving intervention emerges as being, in line with prospect theory, inversely proportional to the magnitude of the threat, rather than being determined by the absolute number of lives the intervention can save ^18, 19^.

We argue that the inability to relate emotionally to the loss of a relatively small amount of health by very large numbers, compared to the ability to relate to the gain of even a moderate amount for an identified individual – say one QALDay for 30,000 people compared with 1 QALY for one person - is to be treated as a problem to be addressed and overcome at the policy level, not to be automatically accommodated.

## Discussion

The bias in relation to innovation is a corollary of the fundamental one. NICE is charged with objectives other than maximising the increase in public health and among its other obligations is to support innovation. But this turns out to be biased support, in that no support can be provided for the development of technologies that are simply cost-effective. These would include innovations which could improve population health by being less costly and less effective – such as SWA in Figure 1, or ones further to the east of the SW quadrant, including the ones that would fall under a kinked ICER, or meet the MAD test of Kent *et al.* No innovation in the SW quadrant can meet the filter test of clinical effectiveness administered prior to the test of cost-effectiveness. So while NICE has a remit to support the adoption of innovative new technologies, in practice the support is confined to those that will cost more.

Eckerman and Pekarsky have exposed the weaknesses of the current NICE procedures as contributions to improved allocative efficiency in the NHS ^6^. Unless the disinvestment to fund a new technology occurs in the least cost-effective activity in the whole service, then allocative efficiency will not be improved as much as it could be, and indeed is quite likely to be reduced.

This is indisputable conceptually, but even more important, the existence of the missing knowledge of the actual shadow price would pose extreme difficulties for NICE. As Paulden and colleagues point out.

The use of thresholds based upon Eckermann and Pekarsky’s proposals by reimbursement bodies would likely result in fewer new technologies being adopted by public healthcare systems. To the extent that this might provide opportunity for resources to be reallocated into more efficient existing health services, this ought to be welcomed. Nevertheless, the implied consequence that technologies be rejected on the basis that there is a preferred option, but one that cannot be implemented, may be a bridge too far for most reimbursement bodies. This is particularly true for NICE, which has a remit, amongst other things, to support the adoption of innovative new technologies, and which operates in a political environment where the adoption of such a low threshold might be untenable. ^7^ p.318

Perhaps the supreme irony in this respect is that 'innovations' falling in the SW quadrant are in fact daily occurrences in most health services, though the denial of this reality, seen as necessary for political survival, persists. The problem is not merely that such SW innovations are disguised or denied - we see them as essential to the future of any National Health Service - but that they occur disproportionately in politically vulnerable areas of the service and with no consideration, even informal, of whether they were cost-effective at any threshold. For example, reducing the numbers of staff such as nurses, saves money at the expense of the effectiveness/quality of the service. The common pretence is that such a change falls in the SE quadrant, usually on its western border where no loss is suffered, few having the audacity to claim it actually increases effectiveness. This fools only those who wish to be fooled, who may or may not include the managers responsible, whose careers depend on delivering apparently SE changes within shrinking budgets.

None of this is in any way intended to discourage the search for and implementation of SE innovations. But the much publicised LEAN ones, which involve working smarter not harder, may well fall in the SW quadrant, as well as the SE, and still represent increased cost-effectiveness ^20^.

There is, also ironically, an excellent example of NICE implementing a SW innovation in its own operations: its introduction of the cheaper Single Technology Appraisal, where the manufacturer is responsible for the analysis and an independent team is paid only to critique it, not conduct a full-scale Multi Technology Assessment using all appropriate comparators ^21^. It seems politically unacceptable to admit that this is undoubtedly reducing the quality of the appraisal, even though the reduction could conceivably be relatively small and the cost saving large, thereby releasing resources for other uses - the essence of the SW argument.

It is not as if the key underlying issue is not well recognised by Claxton and colleagues ^22^

One explanation for… ‘Acceptance creep’ (in the NICE appraisal process) is that the broad selection of stakeholders who contribute to the NICE process excludes a key constituency: those unidentified NHS patients who bear the true opportunity costs of NICE decisions. NICE undoubtedly faces extensive pressure from the direct beneficiaries of a positive recommendation, including manufacturers, the patients who might benefit and their clinicians. Indeed, these stakeholder groups have, quite appropriately, become an important part of the appraisal process. However, without institutional leadership to ensure balance, there is much less pressure to take full account of the likely impact on other NHS patients. The most recent evidence and the nature of the recent proposals suggests that NICE is not providing sufficient leadership and is failing to uphold this critical responsibility to all NHS patients. ^1^. p.2

The evidence suggests that more harm than good is being done, but it is the unidentified and unrepresented NHS patients who bear the true (health) opportunity costs. Although finding reasons to approve new drugs is undoubtedly politically expedient, this cannot be ethically literate, because the interests of NHS patients, whether they are identifiable or not, are just as real and equally deserving of the type of care and compassion that can be offered by a collectively funded health care system. It is to be hoped that NICE will begin to place the unidentified NHS patients who bear the real opportunity costs at the heart of its deliberative process; especially as it reconsiders how other attributes of benefit might be taken into account. ^1^ p.6

The question is whether they will acknowledge that their arguments require at least noting the elephant in the SW corner of the policy room, and suggesting that it cannot be ignored by those at the table if they wish to pursue cost-effectiveness in an unbiased way. The efforts to justify this censoring of cost-effectiveness, albeit well-intentioned in many cases, unfortunately coincide with the material interests of powerful stakeholders, commercial, professional and political, which are not always aligned with those of the citizens. Independent analysts need to ensure that they are not colluding, and, to avoid this accusation, should state explicitly that they have been told to not go SW.

## Conclusions

The SW argument is simply that, given cost-effectiveness is the most important route to maximising group level health gain, not applying it logically and consistently in the SW as well as the NE quadrant is a clear breach of the opportunity cost-effectiveness principle and its underlying justification. While implementing the principle requires many lower-level and difficult decisions ^23^, these must not be allowed to undermine the case for using it.

If one wants to reject cost-effectiveness as a principle, that is clearly possible. But distorting it, either by refusing to consider intervention in the SW quadrant or imposing different requirements (different threshold, or different demands regarding displacement impact) undermines the case for employing it at all, whether on efficiency or ethical grounds, or both. The task is to have SW innovations legitimated and discussed and evaluated as transparently as those in the NE.

The local and global consequence of rejecting the SW argument is that there is little or no incentive to develop interventions that are cost-effective by being cheaper but less effective - especially ones that would be considerably cheaper but only slightly less effective at the individual level. These would include many non-pharmacological interventions, including such things as health literacy promotion, decision support for medication adherence, or simple home care.

It is hard to convince people that making things better in one part of a system does not necessarily make them better overall, in fact often worse. So the ubiquitous mantra of ‘lowering costs without compromising quality’ ^24^ needs to be seen as part of the problem as well as part of the solution. There is a parallel to the ‘tragedy of the commons’ here ^25^.

The healthy, selfish Rawlsian concerned only with themselves and their relatives should consider the opportunity costs of all policy decisions as if they were an anonymous other and therefore support unbiased application of the cost-effectiveness principle.

*Caveat emptor* must be the message to potential NICE buyers, particularly in low or middle income countries ^26^, but certainly not only in them.

## Appendix

**Text adapted from 1. This text has been modified to refer to the NE quadrant of Figure 1 and includes additional labels: NHB, NEA, and NEB.**

“As [ Figure 1] illustrates, CEA effectively becomes an analysis of net health benefits [(NHBs)]: does the health gain from the new intervention outweigh the health decrements associated with the displacement of existing services necessary to fund it? [ Figure 1] shows the incremental costs and QALYs associated with a new intervention [NEA] relative to a comparator (the latter being shown at the origin). The new intervention generates 2 additional QALYs per patient and, at price P1, imposes an additional £20,000 per patient; the ICER is, therefore, £10,000 per QALY gained. At a threshold of £20,000 per QALY, the additional cost of £20,000 per patient translates into a decrement of 1 QALY (the distance between the y-axis and the threshold). This is because the threshold indicates the additional cost that needs to be imposed on the NHS budget in order to displace services that result in 1 QALY being forgone. Therefore, at that price, there is a net health gain of 1 QALY per patient (2 gained from the new intervention and 1 forgone through displacement). At a price of P2, the additional cost per patient of the new intervention [NEB] is £40,000 and the net health gain is 0: the 2 additional QALYs from the new intervention are the same as the QALYs forgone through displacement. At the highest price of P3, the adoption of the new intervention [NEC] would actually result in a net health decrement of 1 QALY as it generates fewer QALYs (2) than are forgone (3).” 1, pp. 3–4.

**Text adapted from 1. This text has been modified to refer to the SW quadrant of Figure 1 and includes additional labels: NHB, SWA, and SWB.**

“As [ Figure 1] illustrates CEA effectively becomes an analysis of net health benefits [(NHBs)]: does the health gain from the new intervention outweigh the health decrements associated with the displacement of existing services necessary to fund it? [ Figure 1] shows the incremental costs and QALYs associated with a new intervention relative to a comparator (the latter being shown at the origin). The new intervention [SWA] generates ^3^ additional QALYs per patient and, at [saving (negative price)] P1, [saves] £20,000 per patient; the ICER is, therefore, £10,000 per QALY gained. At a threshold of £20,000 per QALY, the [reduced] cost of £20,000 per patient translates into [an increment] of 1 QALY (the distance between the y-axis and the threshold). This is because the threshold indicates the [reduced] cost […] imposed on the NHS budget [as a result of the 2 QALYs] being forgone. Therefore, at that price, there is a net health gain of 1 QALY per patient ( 3 gained from the new intervention and 2 forgone through displacement). At a [saving (negative price)] of P2, the cost [reduction] per patient of the new intervention [SWB] is £40,000 and the net health gain is 0: the 2 additional QALYs from the new intervention are the same as the QALYs forgone through displacement. At the [lowest saving (negative price)] of P3, the adoption of the new intervention [SWC] would actually result in a net health decrement of 1 QALY as it generates fewer QALYs ^1^ than are forgone (2).” 1, pp. 3–4.
