# Supplementary material for: Caveat emptor NICE: biased use of cost-effectiveness is inefficient and inequitable
Source: F1000Res. 2015 Oct 16;4:1078. [Version 1] doi: 10.12688/f1000research.7191.1 (PMC5074351; doi:10.12688/f1000research.7191.1)
Supplement: Peer review history

# Peer review: Referee response for version 1

**DOI:** 10.5256/f1000research.7747.r17091

**Published:** 2016-10-19

**Authors:** Jes Søgaard

**Affiliations:** 1 Department of Clinical Epidemiology, Aarhus University, Aarhus, Denmark; 2 Director of Documentation & Quality, Danish Cancer Society, Copenhagen, Denmark

I like the paper: “Caveat Emptor NICE: biased use of cost-effectiveness is inefficient and inequitable” by Jack Dowie *et al* and I have followed it on the way to this version. I think they have a logical point and they argue their case well. I cannot judge whether they miss some arguments against the SW case.

 I have two queries that the authors and/or editors might consider to be relevant for some thought.

1. The kinkiness issue is discussed at some length on page 5, but to me it is unclear whether it is an ethical and logical issue, as the authors conclude (referring to an earlier Dowie paper that I have not read) or whether it is an empirical issue i.e. whether kinkiness should reflect the WTP/WTA ratio “in society”, i.e. take loss aversion into account. In the analysis they state “We maintain that the principle of cost-effectiveness requires that they be the same and that no logical or ethical case can be made for any kinked ICER in a public system” and in the Discussion it seems that the authors accept the MAD principle which could imply kinked ICERs in the plane quadrants.
2. The other point is more practical – how would you administer SW cases. In the NE quadrant you document clinical efficacy and have some cost estimates to estimate ICERs, with cost data from typically secondary endpoints supplemented with observational cost data. In the SW quadrant, should you document cost savings, i.e. have costs as the primary end points for strong cost saving evidence? Should we/authorities encourage pharmaceutical and other industries to develop cost saving drugs and devices with inferior health effects? For example accept inferior generics – if cost savings are large enough? Or is the SW quadrant more relevant with the health providers cost saving ‘innovations’ when third party payers demand cost controls and budget cuts? What would the SW quadrant cases be?

Besides these two queries I think the paper is ready to be indexed.

I have read this submission. I believe that I have an appropriate level of expertise to confirm that it is of an acceptable scientific standard.

# Peer review: Referee response for version 1

**DOI:** 10.5256/f1000research.7747.r10845

**Published:** 2015-10-27

**Authors:** Jeffrey Braithwaite

**Affiliations:** 1 Centre for Healthcare Resilience and Implementation Science, Australian Institute of Health Innovation, Macquarie University, Sydney, NSW, Australia

There are times when health economics articles are dense and jargon laden. There are other times when health economics articles seem to say what common sense suggests anyway.

This article is neither of these. It speaks with logic, authority and a great deal of wisdom about the importance of potential bias in the UK’s National Institute for Care and Health Excellence (NICE) and its approach to cost-effectiveness. The authors make the case that at the end of the day, “while NICE has a remit to support the adoption of innovative new technologies, in practice the support is confined to those that will cost more.” The authors want NICE and by extension anyone involved in this sort of appraisal not to omit to consider innovations and technologies that are simply cost-effective (as compared to those that are firstly determined to be clinically effective, and then assessed for cost-effectiveness).

Without going into the detailed economic and conceptual arguments, which is of less interest to general readers, it is surely incumbent on policymakers to review this argument and take notice of it, or refute it. Either way, we should hear more about the potential of NICE to refine how it enables cost-effectiveness and innovation. Given the role NICE plays nationally and internationally, this is by no means a trivial request.

I have read this submission. I believe that I have an appropriate level of expertise to confirm that it is of an acceptable scientific standard.
